# Characterizing Promoter and Enhancer Sequences by a Deep Learning Method

**DOI:** 10.3389/fgene.2021.681259

**Published:** 2021-06-15

**Authors:** Xin Zeng, Sung-Joon Park, Kenta Nakai

**Affiliations:** ^1^Department of Computational Biology and Medical Science, The University of Tokyo, Kashiwa, Japan; ^2^Human Genome Center, The Institute of Medical Science, The University of Tokyo, Tokyo, Japan

**Keywords:** promoter, enhancer, bidirectional transcript, convolutional neural network, gene regulation

## Abstract

Promoters and enhancers are well-known regulatory elements modulating gene expression. As confirmed by high-throughput sequencing technologies, these regulatory elements are bidirectionally transcribed. That is, promoters produce stable mRNA in the sense direction and unstable RNA in the antisense direction, while enhancers transcribe unstable RNA in both directions. Although it is thought that enhancers and promoters share a similar architecture of transcription start sites (TSSs), how the transcriptional machinery distinctly uses these genomic regions as promoters or enhancers remains unclear. To address this issue, we developed a deep learning (DL) method by utilizing a convolutional neural network (CNN) and the saliency algorithm. In comparison with other classifiers, our CNN presented higher predictive performance, suggesting the overarching importance of the high-order sequence features, captured by the CNN. Moreover, our method revealed that there are substantial sequence differences between the enhancers and promoters. Remarkably, the 20–120 bp downstream regions from the center of bidirectional TSSs seemed to contribute to the RNA stability. These regions in promoters tend to have a larger number of guanines and cytosines compared to those in enhancers, and this feature contributed to the classification of the regulatory elements. Our CNN-based method can capture the complex TSS architectures. We found that the genomic regions around TSSs for promoters and enhancers contribute to RNA stability and show GC-biased characteristics as a critical determinant for promoter TSSs.

## Introduction

Traditionally, promoters are defined as DNA regions where transcription is initiated (Lenhard et al., 2012; Haberle and Stark, 2018). The promoters include specific DNA motifs where transcription factors (TFs) and their complexes can access (Hudson and Quail, 2003). On the other hand, enhancers are defined as DNA regions that amplify transcription initiation by directly interplaying with their target promoters (Blackwood and Kadonaga, 1998). Likewise, the enhancer sequences, distal from their target promoters, contain DNA motifs that act as binding sites for TFs and cofactors. These historical definitions are dichotomic, which means that promoters and enhancers are distinct regulatory elements. However, what factors determine the promoter and enhancer activities remains unclear.

Recently, researchers have found that transcription occurs in both the sense and antisense directions of promoters and enhancers, associating with the alternative transcription start sites (TSSs) (Andersson et al., 2014). Surprisingly, promoters give rise to stable mRNAs in the sense direction and produce unstable RNAs in the upstream antisense direction, while enhancers give rise to unstable enhancer RNAs in both directions (Weingarten-Gabbay and Segal, 2014). To explain the different RNA stability in the sense and antisense directions of promoters, the “U1-PAS axis” model has been proposed (Almada et al., 2013): the 5′ splice site (SS5) motif is enriched at the downstream of TSSs of stable transcripts but is depleted at TSSs of unstable transcripts, and vice versa for the PAS motif. Nonetheless, a hidden Markov model incorporating these motifs predicted the transcript stability at a relatively low accuracy (63%) (Core et al., 2014). As another approach, a support vector machine (SVM) with hexamer nucleotides improved the separation of promoters from enhancers, identified by the FANTOM consortium (AUC 0.86) (Colbran et al., 2019). However, the SVM could not find the sequence features that precisely determine the classification.

In this study, we developed deep learning (DL)-based models to classify promoters and enhancers by incorporating the convolutional neural network (CNN) method. We performed systematic experiments on these datasets to reveal how the CNN architecture, in particular convolutional filter size and max-pooling size, influences the performance of the models. Furthermore, to characterize TSS architectures that are indispensable for the distinctive regulatory activities, we employed the saliency map (Simonyan, 2013), extracting the impactful features.

## Materials and Methods

### Preparing Promoter and Enhancer Datasets

For building a DL model with CAGE (Cap Analysis Gene Expression) data, we prepared the enhancer and promoter dataset defined by the previous study (Colbran et al., 2019), including 38,538 enhancers and 27,227 promoters. These elements have been defined as flanking 300 base pair (bp) regions for each midpoint between the bidirectional CAGE peaks.

We downloaded the bidirectional TSSs at K562 cells from the previous study (Core et al., 2014) that used the combination of GRO-seq (Global Run-On sequencing) and CAGE TSSs; 1,331 stable-stable (SS) pairs, 1,884 unstable-stable (US) pairs, and 4,978 unstable-unstable (UU) pairs. Because the typical bidirectional TSS of enhancers is UU and that of promoters is US (Weingarten-Gabbay and Segal, 2014), we used US and UU datasets and generated the flanking 250-bp regions for each midpoint between the bidirectional TSS (hereinafter referred to as US_UU dataset).

### Implementing CNN

Our CNN models take an input matrix that consists of a one-dimensional one-hot-encoded sequence with four channels. For instance, each nucleotide in the input DNA sequence is represented as *A* = (1, 0, 0, 0), *C* = (0, 1, 0, 0), *G* = (0, 0, 1, 0), and *T* = (0, 0, 0, 1). Therefore, an input DNA sequence of length *l* (*l*=500 in US_UU dataset and *l*=600 in CAGE dataset) is converted into an *l*=4 matrix.

The model processes the input matrix with two convolutional layers, a fully connected hidden layer and a fully connected output layer with one neuron that has sigmoid activations for binary classification. The first convolutional layer employs 30 filters (i.e., the number of filters) each with a size of 19 (i.e., filter size) and a stride of one. The second convolutional layer employs 128 filters each with a size of 5 and a stride of one. All convolutional layers incorporate zero-padding to achieve the same output length as the inputs and are activated by a rectified linear unit (ReLU), which replaces negative values with zero. Each convolutional layer is followed by a max-pooling layer with window size and stride that are equal. The product of the two max-pooling window sizes is equal to 100. For example, if the first max-pooling layer has a window size of two, then the second max-pooling window size is 50. This constraint ensures that the number of inputs to the fully connected hidden layer is the same across all models. The fully connected hidden layer employs 512 units with ReLU activation.

The dropout layer, which is a common regularization technique for deep neural networks, is applied during training after each convolutional layer and the fully connected hidden layer with dropout probability of 0.5. The dropout probability was chosen empirically (data not shown). All models were trained via mini-batch stochastic gradient descent algorithm with mini-batch size of 30 sequences for 40 epochs. The parameters are learned on each mini-batch set by minimizing the cross-entropy loss function *L* given as,

$$\text{L}=-\frac{1}{N}\,\sum_{i=1}^{N}y_{i}\times \log z_{i}\,\left(1-y_{i}\right)\times \text{log}\,\left(1-z_{i}\right),$$

where *z*_*i*_ is the true label (either 0 or 1) for training data in the *i*-th sequence in each mini-batch, *y*_*i*_ is the predicted label produced by a sigmoid function in the *i*-th sequence in each mini-batch, and *N* is the mini-batch size. The loss function *L* is updated by Adam algorithm (Kingma and Ba, 2015) with the recommended parameters: learning rate of 0.0003 and learning rate decay of 1e-6.

We carried fivefold cross-validation to test the generalization of the models and evaluated the models with the average area under the receiver-operator-characteristic (AU-ROC).

### Implementing Saliency Algorithm

Saliency map has been derived from the concept of saliency in images that the most important pixels are highlighted (Simonyan, 2013). Given a well-trained CNN model, the saliency map visualizes the change of output probability that is caused by slight alterations on each pixel of the input image, which suggests the importance of the pixels: the more varying changes mean the more important pixels. We utilized the saliency algorithm for detecting the most noticeable features in the input data and for improving model explainability.

Concretely, given an input DNA sequence *X* of certain length |*X*|, our CNN model can calculate the score S_c_(*X*)for the class *c* at the output layer using a linear function of the first-order Taylor expansion:

$$S_{c}\,\left(X\right)\approx {\text{w}}^{T}\,\text{X}+\text{b}=\sum_{i=1}^{\left|X\right|}{\text{w}}_{i}\,x_{i}+\text{b},$$

where *w_i* is the weight for the *i*-th nucleotide *x*, and *b* is the bias of the model. The weights represent the importance of each nucleotide in the predictive power of the model. The saliency algorithm alters each nucleotide in the *X* and calculates the weights using a one-step back-propagation in the CNN model, then visualized the weights on the saliency map. More details can be found from the previous studies (Simonyan, 2013; Lanchantin et al., 2017). We normalized the scores by dividing the sum of the scores in each sequence. Furthermore, we defined the important positions in each sequence with the normalized “importance score” larger than 1/|*X*|; 0.002 in the US_UU dataset and 0.00167 in the CAGE dataset, which reflects the fact that each nucleotide in a random sequence will contribute equally.

### Bioinformatics Analysis

We calculated the GC content for a region of interest as *GCContent*=(*GC*)/(*ATGC*). For comparison purposes, we prepared the results of the SVM model for the CAGE dataset from the previous study (Colbran et al., 2019) and trained SVM models for the US_UU dataset using the encoding approach in that study; the input vector to SVM is formed as the frequencies of all the possible combinations of 6-bp-nucleotides, 4096 dimensions, in each input DNA sequence. We trained the SVM models by grid search with *C* (from −3 to 1) and γ (from −13 to −1) and selected a model that has the highest AU-ROC. We implemented our model using Tensorflow 2.1.0 of the programming language Python 3.7 on the computer that installed the Intel Core i5 (3.4 GHz, 4cores) and 32GB of main memory. The training of a US_UU model requires 13–15 min but depends on the hyperparameter setting. The training of a CAGE model requires approximately 170 min depending on the hyperparameter setting. We used the R language for processing and visualizing.

## Results

### Overall Structure of the Datasets

**FIGURE 1:**
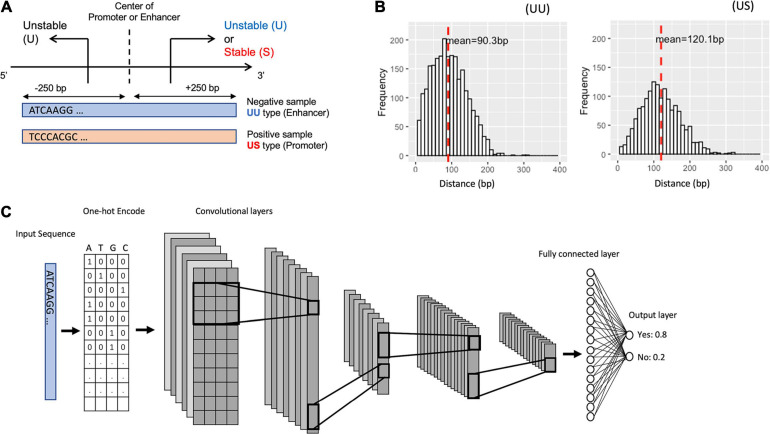
Schematic representation for bidirectional TSSs addressed in this study and for the structure of the proposed deep learning model. **(A)** The definition of the promoter and enhancer TSS pairs. **(B)** Histogram showing the distance distribution between the sense and the antisense TSSs. **(C)** The overall structure of our CNN model. TSS, transcriptional start site; CNN, convolutional neural network.

To characterize the promoter and enhancer regions, we first prepared publicly available annotations, which have been based on GRO-seq (Core et al., 2008) and CAGE tags (Harbers and Carninci, 2005). The GRO-seq data provide information on the transcript stability, while the CAGE tags generate TSS clusters at enhancer and mRNA TSSs. By combining these data, we could define the bidirectional UU TSS pairs and US TSS pairs, which correspond to enhancer and promoter regions (Figure 1A). The distances between UU and US TSS pairs were 91 and 120-bp on average respectively with 400-bp in maximum (Figure 1B). We decided to use the flanking 250-bp regions for each from the center of TSS pairs to cover the downstream regions of TSSs. In addition, we prepared the CAGE-defined 600-bp regions for bidirectional enhancer and promoter TSSs (Colbran et al., 2019) in which the stability of transcripts was not considered (Table 1).

**TABLE 1:** Contents of the dataset used in this study.

**Dataset**	**Promoter**	**Enhancer**	**Train**	**Test**
CAGE	27,227	38,538	59,189 (90%)	6,576 (10%)
US_UU	1,884	4,978	6,176 (90%)	686 (10%)

We divided the datasets into a training set (90%) and a hold-out test set (10%). To select the best CNN model, we applied fivefold cross-validation during the training process, which splits the dataset into five groups, fits a model with four groups, and evaluates the model with the remaining one group. To better test the robustness of the model, we also evaluate the models with an independent test set for each cross-validation, and we use the mean AU-ROC of the validation set and the hold-out test set as the metrics of the models. We fed the DNA sequences of the promoter regions (positive samples) and of the enhancer regions (negative samples) into the CNN models by one-hot encoding (Figure 1C).

### Tuning Hyperparameters for the Convolutional Layer of CNN Models

Previous studies have suggested that the first convolutional layer learns the representations of sequence motifs (Kelley et al., 2016; Quang and Xie, 2016). Here, we explored how the hyperparameters in the first layer contribute to the performance of the models. We used the hyperparameter setting framework (Koo and Eddy, 2019).

We systematically modified the max-pool size, while keeping all other hyperparameters fixed, including the number of filters and the filter sizes in the first and the second layers (see section “Materials and Methods”). To minimize the influence of architecture on classification performance, we coupled the max-pool size between the first layer and the second layer such that their products are consistent. This ensures that the size of inputs into the fully connected hidden layer of CNN models is equal. The max-pool sizes we employed are (1, 100), (2, 50), (4, 25), (10, 10), (25, 4), (50, 2), and (100, 1), where the numbers represent (the first layer and the second layer). Note that each CNN model is denoted with the first max-pool size for simplifying, such as CNN-1 for (1, 100), CNN-2 for (2, 50), and so on.

We compared the AU-ROC curve across the two datasets (Table 2). We found that the models employing the max-pool size 10 reach the highest performance. This suggests that the middle size of the max-pooling can retain more information in the data, which is helpful to capture the ground truth motifs. In contrast, the models employing the small (=1) or large max-pool sizes (=100) showed worse performances. These results imply that a large down-sampling size either in the first layer or the second layer causes distortion, which limits the ability of models to learn motif representations in the enhancers and promoters in deeper layers.

**TABLE 2:** Performance of CNN models with different hyperparameters.

**Model**	**The first layer**			**The second layer**			**AUC**	
	**#. Filters**	**Filter size**	**Max-pool size**	**#. Filters**	**Filter size**	**Max-pool size**	**US_UU**	**CAGE**
CNN-1	30	19	1	128	5	100	0.903 ± 0.014	0.899 ± 0.007
CNN-2	30	19	2	128	5	50	0.910 ± 0.017	0.905 ± 0.009
CNN-4	30	19	4	128	5	25	0.910 ± 0.018	0.914 ± 0.126
CNN-10	30	19	10	128	5	10	0.915 ± 0.020	0.917 ± 0.014
CNN-25	30	19	25	128	5	4	0.910 ± 0.018	0.913 ± 0.015
CNN-50	30	19	50	128	5	2	0.910 ± 0.016	0.908 ± 0.012
CNN-100	30	19	100	128	5	1	0.901 ± 0.012	0.898 ± 0.005
CNN-10(60)	60	19	10	128	5	10	0.930 ± 0.018	0.925 ± 0.014
CNN-10(90)	90	19	10	128	5	10	0.932 ± 0.018	0.932 ± 0.015
CNN-10(120)	120	19	10	128	5	10	0.931 ± 0.017	0.934 ± 0.017
CNN_9_-10(90)	90	9	10	128	5	10	0.928 ± 0.021	**0.935 ± 0.016**
CNN_29_-10(90)	90	29	10	128	5	10	**0.934 ± 0.021**	0.931 ± 0.016

*Bold value represents the maximum in the column.*

For investigating the influence of the number of filters in the model performance, we increased the number of CNN-10 from 30 to 60, 90, and 120, which denoted as CNN-10(60), CNN-10(90), and CNN-10(120). Upon training each of these models, we found that increasing the number of filters improves the performances (Table 2). However, overparameterization such as CNN-10(120) seems less effective, which is consistent with previous studies (Koo and Eddy, 2019). These results suggest that overparameterizing the number of filters results in more filters that do not learn any motif representations.

Next, for testing the influence of the filter size of each filter in the first layer, we created two new CNN-10(90) models, CNN_9_-10(90) and CNN_29_-10(90) that employ 9 and 29 for the first-layer filter size. As a result, all the models share similar performances (Table 2), which suggests that motif representations are not very sensitive to the first-layer filter size.

### Comparing the Proposed Model With SVM

In order to compare our model with the SVM of hexamer nucleotides (Colbran et al., 2019), we selected CNN-10(90), one of the best models that less vary the performance. We tuned the hyperparameters of SVM by applying the grid search.

As a result, both the SVM and the CNN model exhibited higher AUC values overall, which suggests that the feature of DNA sequences is the primary choice for distinguishing promoters and enhancers (Table 3). Notably, the CNN model showed better performance than the SVM model. This result implies the importance of higher-order sequence information for distinguishing these regulatory elements more precisely.

**TABLE 3:** Comparison of the proposed model with SVM.

**Dataset**	**Model**	**Performance (AUC)**	
		**Train**	**Test**
CAGE	SVM	0.86	0.87
	CNN-10(90)	0.95	**0.91**
US_UU	SVM	0.81	0.82
	CNN-10(90)	0.96	**0.89**

*Bold value represents the maximum in the column.*

### Detecting Important Sequence Positions in the Classification

Given an input DNA sequence for testing, we calculated the gradient of the output probability by modifying the nucleotides in the input sequence: the greater the predicted probability changes at a position, the more important the nucleotide at this position. Using the saliency algorithm (Lanchantin et al., 2017), we defined the probability changes as a normalized “importance score” *S*.

**FIGURE 2:**
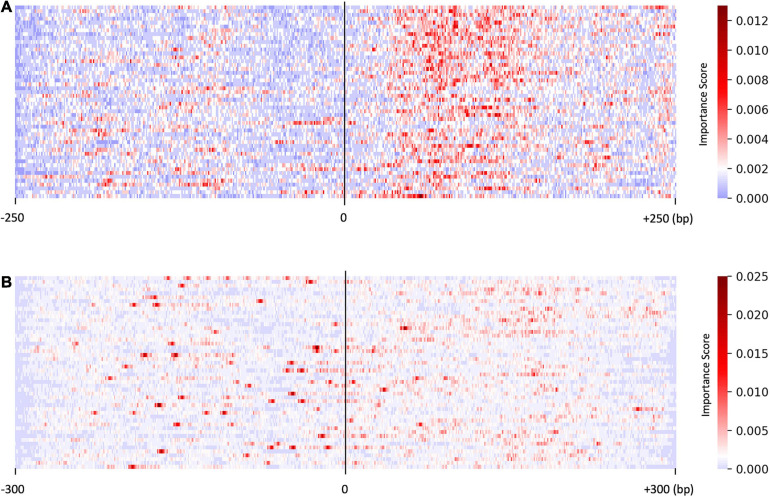
Results of the saliency map showing the importance score of each nucleotide in the top 50 predicted promoter regions with US_UU dataset **(A)** and CAGE dataset **(B)**. The *x*-axis represents relative positions from the midpoint between the bidirectional transcriptional start site (TSS). The color scale corresponds to the normalized importance score; US, unstable-stable TSS pair; UU, unstable-unstable TSS pair.

We investigated the distribution of the score *S* by focusing on the sequences predicted with higher probability (>0.95, resulted in 50 sequences). As shown in Figure 2A, the saliency map for the predicted US sequences in the US_UU dataset indicated that +20–120 bp regions strongly contribute to the classification. This result is consistent with a previous study, in which the motifs located downstream of TSSs affect RNA stability (Almada et al., 2013). In contrast, the saliency map for the predicted promoters in the CAGE dataset (Figure 2B) visualized that the important nucleotides for the classification of CAGE-defined promoters and enhancers are rather scattered than those observed in Figure 2A.

These results suggest that there exist substantial sequence differences between promoters and enhancers and indicate that the promoter architecture differs in regulating stable transcripts and promiscuous transcripts.

### Characterizing Sequence Compositions in the Regulatory Elements

It has been known that CpG island is an important feature of promoters but not of enhancers (Andersson et al., 2014). To confirm whether our models learned this distinction or not, we calculated GC contents of the promoter and enhancer regions, as well as those of the important positions predicted by the saliency maps.

**FIGURE 3:**
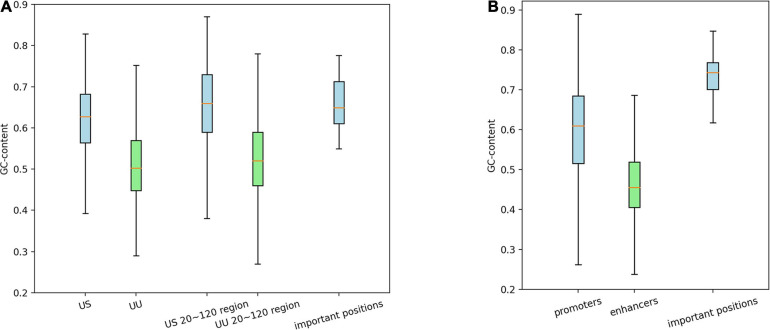
Distribution of GCs in the regulatory regions and in the predicted important positions, which presents GC-biased characteristics in US TSS pairs **(A)** and in CAGE-defined promoters **(B)**. G, guanine; C, cytosine; TSS, transcriptional start site; US, unstable-stable TSS pair.

As shown in Figure 3A, on average, G and C accounted for 63% in the US TSS pairs, whereas for 50% in the UU TSS pairs, which is consistent with the observation in the previous studies (Fenouil et al., 2012; Andersson et al., 2014). For the +20–120 bp regions, this trend where the US TSS pairs present higher GC content than UU TSS pairs was also observed: 67% in US and 51% in UU. The important positions (>0.002 in the score *S*) in the saliency map (Figure 2A) showed 68%. Similarly, as shown in Figure 3B, the CAGE-defined promoters showed higher GC content (61%) than the CAGE-defined enhancers (45%). Remarkably, the important positions (>0.00167 in the score *S*) presented 74% (Figure 2B).

These results suggest that the CNN model achieves high predictive performance by incorporating the distinctive GC content in the regulatory regions, which is a potential determinant for promoter and enhancer activities.

## Discussion

In this study, we sought to decipher the characteristics of bidirectional TSS architectures of promoters and enhancers. In this regard, previous studies have focused on the SS5 and PAS motifs at the downstream of TSSs that are potentially associated with RNA stability (Almada et al., 2013; Wu and Sharp, 2013). Due to the use of such simple and limited motif patterns, the previous model was not enough to explain the differences (Core et al., 2014). Here we extended the focused regions by developing a CNN-based DL model and estimating the importance of each nucleotide in the regions.

We tested the CNN models with different sets of hyperparameters and found that the max-pool size and the filter number affect the performance, rather than the size of each first-layer filter. By employing a set of tuned parameters, we performed the proposed model and an SVM-based model for reference. The results suggested that the sequences around the bidirectional TSS regions possess information to distinguish promoters from enhancers. Compared to the SVM-based model employing k-mer patterns, the CNN model presented better predictive performance, suggesting that the high-order sequence features are indispensable to precisely characterize these regulatory regions.

Interestingly, the promoter regions presented a different distribution of key nucleotides for the classification. Particularly, when we focused on the promoters regulating genes that produce stable transcripts, we found that approximately 100-bp continuous sequences have GC-biased characteristics, which is a potential determinant for promoter and enhancer activities.

Although we could not observe statistically significant TF-binding motifs within the important regions, there may have signatures that specify the crosstalk with other genetic and epigenetic factors to be studied as future work.

## Data Availability Statement

The original contributions presented in the study are included in the article/Supplementary Material, further inquiries can be directed to the corresponding author.

## Author Contributions

KN conceived of and designed the study. XZ, SJP, and KN designed, performed all the analyses, and drafted the manuscript. All authors read and approved the final manuscript.

## Conflict of Interest

The authors declare that the research was conducted in the absence of any commercial or financial relationships that could be construed as a potential conflict of interest.

## Supplementary Material

Supplementary Figure 1 The ROC (Receiver Operating Characteristic) curves of CNN-10(90) model with **(A)** US_UU dataset and **(B)** CAGE dataset calculated by fivefold cross-validation.
