# Supplementary figures and images for: Characterizing Promoter and Enhancer Sequences by a Deep Learning Method
Source: Front Genet. 2021 Jun 15;12:681259. doi: 10.3389/fgene.2021.681259 (PMC8239401; doi:10.3389/fgene.2021.681259)

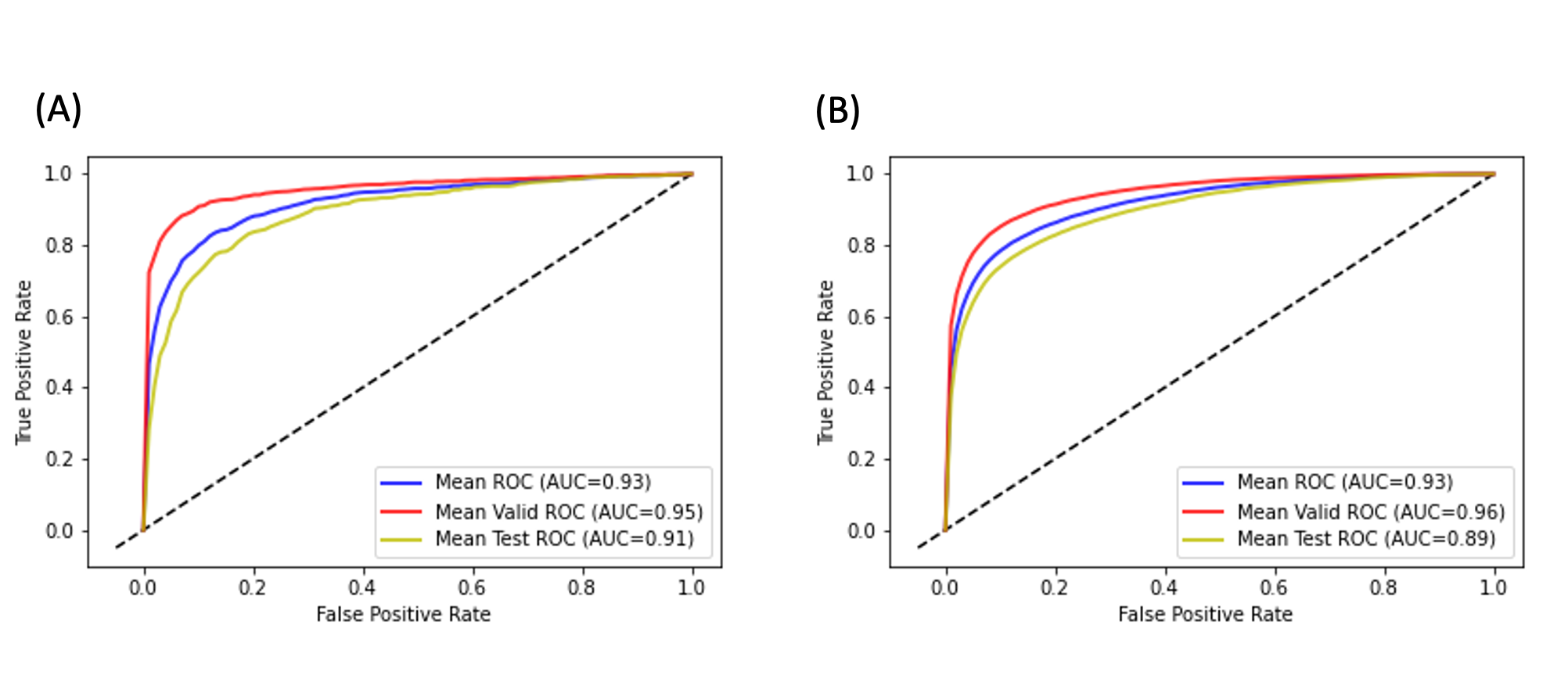

Supplement: Supplementary Figure 1 — The ROC (Receiver Operating Characteristic) curves of CNN-10(90) model with (A) US_UU dataset and (B) CAGE dataset calculated by fivefold cross-validation. [file Image_1.PNG]
